# Technology-driven approaches for meiosis research in tomato and wild relatives

**DOI:** 10.1007/s00497-022-00450-7

**Published:** 2022-09-23

**Authors:** Sander A. Peters, Charles J. Underwood

**Affiliations:** 1 Business Unit Bioscience, Cluster Applied Bioinformatics, Wageningen University and Research, Droevendaalsesteeg 1, 6708 PB Wageningen, The Netherlands; 2 Department of Chromosome Biology, Max Planck Institute for Plant Breeding Research, Carl-von-Linné-Weg 10, 50829 Cologne, Germany

**Keywords:** Tomato, Meiosis, Crossover, Genomics, Genome editing, Plant hybrids

## Abstract

Meiosis is a specialized cell division during reproduction where one round of chromosomal replication is followed by genetic recombination and two rounds of segregation to generate recombined, ploidy-reduced spores. Meiosis is crucial to the generation of new allelic combinations in natural populations and artificial breeding programs. Several plant species are used in meiosis research including the cultivated tomato (*Solanum lycopersicum*) which is a globally important crop species. Here we outline the unique combination of attributes that make tomato a powerful model system for meiosis research. These include the well-characterized behavior of chromosomes during tomato meiosis, readily available genomics resources, capacity for genome editing, clonal propagation techniques, lack of recent polyploidy and the possibility to generate hybrids with twelve related wild species. We propose that further exploitation of genome bioinformatics, genome editing and artificial intelligence in tomato will help advance the field of plant meiosis research. Ultimately this will help address emerging themes including the evolution of meiosis, how recombination landscapes are determined, and the effect of temperature on meiosis.

## Introduction

Meiosis is a cell division that takes place during reproduction and leads to the generation of recombined, ploidy-reduced spores in most sexual eukaryotic organisms (Mercier et al. 2015). During the first meiotic division replicated chromosomes pair and synapse with their homologous partners which involves the formation of a “proteinaceous bridge” between the homologous chromosomes called the synaptonemal complex (SC) (Wang and Copenhaver 2018). Linked to this process, chromosomes are targeted by programmed DNA double strand breaks and homologous chromosomes genetically recombine with one another. Recombination between the homologues can involve large (megabase pair-scale) reciprocal exchanges of genetic information—meiotic crossovers (CO)—or the copying of small (25–50 base pair-scale) patches of sequence from one homologue to another, which is commonly called non-crossover (NCO) repair or gene conversion (GC) (Wijnker et al. 2013; Rommel Fuentes et al. 2020). Meiotic COs can be formed via two major pathways (Wang and Copenhaver 2018). The class I pathway (otherwise known as the ‘ZMM’ or ‘interfering’ pathway) makes up about 85% of CO in Arabidopsis and the class II pathway (otherwise known as the ‘non-interfering’ pathway) makes up about 15% of CO in Arabidopsis (Wang and Copenhaver 2018). After the resolution of COs, and the loss of molecular links between the recombining chromosomes, segregation of homologous chromosomes occurs. In diploid sexual organisms, sister chromatids segregate during the second meiotic division to generate haploid spores (Wang et al. 2021). After meiosis the spores enter gametogenesis and eventually the fusion of male and female gametes, by fertilization, leads to the establishment of genetically unique, diploid, offspring (Dresselhaus et al. 2016).

Like most fundamental plant science, the plant meiosis field has harnessed Arabidopsis as a model system since the 1990s (Albini 1994; Ross et al. 1996), leading to many fundamental insights into the molecular pathways involved in plant meiosis (Mercier et al. 2015; Wang and Copenhaver 2018). Spurred on by the release of the tomato genome (Sato et al. 2012) and rapid implementation of genome editing (Brooks et al. 2014), the cultivated tomato, an important horticultural crop, has re-emerged as a popular plant model in recent years. Here, we argue that the eudicot cultivated tomato (*S. lycopersicum*) and related wild species are highly suitable model systems for plant meiosis research (Box 1).

**Box 1:** **Nine favorable attributes of tomato as a meiotic model**

Large chromosomes (all 12 chromosomes are between 53 and 96 Mbp) that have been extensively characterized cytologically during meiosis
The cultivated tomato (*S. lycopersicum*) can be crossed with it’s 12 closest wild relatives, representing more than 2 million years of genetic divergence, allowing for study of meiosis in wide interspecific hybrids
Medium sized genome (~ 900 Mbp) makes genome sequencing approaches cost-efficient
Time- and cost-efficient pollen nuclei sequencing approaches have been established
High efficiency genetic transformation and genome editing of many inbred and hybrid genotypes is possible
Dwarf, rapid flowering tomato varieties like Micro-Tom are easy to cultivate and can produce flowers 4–5 weeks after sowing
Through precise cultivation and propagation by cuttings, tomato varieties can form flowers all-year-round providing a constant supply of meiotic buds and pollen for experiments or crosses
No recent history of polyploidy and therefore typically low number of gene duplications
Tomato is globally the most valuable crop in the vegetable category and therefore research findings have potential direct applications in tomato breeding and improvement

Tomato has long been recognized as a powerful model system for studying meiotic chromosomes cytologically (Lindstrom and Koos 1931), and due to its horticultural importance was one of the first plant species to have a high density molecular genetic map (Tanksley et al. 1992). During the 1960s, morphological features were identified for distinguishing each of tomato's twelve chromosomes in somatic cells and meiotic pachytene cells (i.e. after the pairing of homologous chromosomes has taken place) (Ramanna and Prakken 1967). In the 1990s recombination nodules, physical structures that can be observed on the central region of the SC that represent future CO events, were counted on tomato pachytene chromosomes (Sherman and Stack 1995). This work showed that euchromatin is most prone to meiotic CO events, while highly reduced CO activity was observed in heterochromatin and absolute absence of meiotic CO was found at kinetochores and chromosome ends (Sherman and Stack 1995). Tomato recombination nodule counting also showed that COs are non-randomly distributed along recombining chromosomes (Sherman and Stack 1995; Lhuissier et al. 2007) providing evidence for CO interference, a well-known phenomenon where the occurrence of one CO reduces the probability of nearby CO events (Hillers 2004). Through the immunocytochemical staining of the DNA mismatch repair protein MLH1 it was conclusively demonstrated that MLH1 marks a subset of tomato COs that are part of the class I/“interfering” CO pathway (Lhuissier et al. 2007). Later work showed that the class II CO pathway in tomato appears to be more active in heterochromatic regions of the tomato genome (Anderson et al. 2014). In parallel to cytological work on recombination in tomato molecular genetic approaches led to an improved genetic map (Shirasawa et al. 2010), which was combined with BAC-FISH results (Szinay et al. 2008) as an important foundation for the original assembly of the tomato genome (Sato et al. 2012). Improved versions of the tomato genome assembly (Hosmani et al. 2019), assemblies of other tomato varieties (Alonge et al. 2021; Rengs et al. 2022) and wild relatives (Bolger et al. 2014; Wang et al. 2020) form an important basis for generating high resolution insights into meiotic recombination in tomato.

New technologies are expanding the possibilities for meiosis research in tomato and other plant species. Through genomics, comprehensive genotyping of recombinant gametes or offspring is now relatively cost-effective, for plant species with small genomes such as Arabidopsis (~ 130 Mbp) and rice (~ 410 Mbp) and even for medium-size plant genomes like tomato (~ 900 Mbp) (Rowan et al. 2015; Rommel Fuentes et al. 2020; Naish et al. 2021; Rengs et al. 2022; Zhou et al. 2022; Zhang et al. 2022). Through genome editing, there are now well-established approaches for making tomato gene knock-outs (Brooks et al. 2014; Čermák et al. 2017) and even the generation of specific point mutations in tomato (Lu et al. 2021) and engineered chromosomes in other species (Schmidt et al. 2020; Schwartz et al. 2020) has become a reality. In addition, developments in super-resolution microscopy, fine-tuned plant cultivation and machine learning suggest that the coming years will allow for the expansion of plant models used in the meiosis field, leading to fundamental and applicable discoveries.

## Genomics enabled approaches for recombination studies in tomato

Tracking and delineation of recombination events in tomato has been carried out through various cytological strategies, as outlined above (Sherman and Stack 1995; Lhuissier et al. 2007; Anderson et al. 2014). Despite the important insights provided by such studies, the low throughput and lack of DNA sequence-based information limits rapid and quantitative measurements of recombination. Through the genotyping of polymorphic genetic markers, the recombination landscape of a segregating population can be determined with high precision. The segregation of genetic markers provides information on recombination frequency and genetic distances that can ultimately be used for genetic linkage map construction. Such analyses have provided further insight into ‘hot’ and ‘cold’ regions’ of recombination, and genomic features determining CO recombination (Fransz et al. 2000; Wijnker et al. 2013; Choi et al. 2013; Aflitos et al. 2014; Demirci et al. 2017, 2018; Underwood et al. 2018; Rowan et al. 2019; Fuentes et al. 2022). In Arabidopsis and Tomato, COs are overrepresented in non-coding regions upstream of the transcriptional start site and downstream of the transcriptional termination site (Wijnker et al. 2013; Choi et al. 2013; Demirci et al. 2017; De Haas et al. 2017). Such regions are enriched with AT-rich sequence motifs in both species. In addition, NCO associated allelic and ectopic GCs detected in tomato confirm that besides CO, GC represents a source for genetic diversity and genome plasticity (Rommel Fuentes et al. 2020). However, there are still numerous gaps of knowledge with respect to CO formation in plants in relation to genetic, epigenetic, and environmental determinants. In this respect DNA and retrotransposon families have recently been shown to be respectively associated with hot and cold spots of meiotic recombination. In Arabidopsis, rice, wheat and potato specific superfamilies of DNA transposons are apparently abundant in recombination-prone regions, which may be explained by nucleosome depletion in DNA transposons (Darrier et al. 2017; Marand et al. 2017, 2019; Choi et al. 2018; Underwood and Choi 2019) In tomato specific transposable elements such as *Sonata*, *Harbinger* and other repeats, were found frequently located near or at synteny junction breaks and have been considered in repeat-mediated repair via homologous and ectopic recombination (Peters et al. 2012). A recent study on historical recombination in cultivated and wild tomato shows that DNA transposons including *hAT-Tip100* and *Stowaway* elements show enrichment in hot spots, whereas most class I retrotransposons including the *Copia* and *Gypsy* element classes are overrepresented in cold spots of recombination (Fuentes et al. 2022). Furthermore, hot and cold spots of recombination have been found to respectively associate with high and low chromatin accessibility in tomato (Chouaref 2021). These observations suggest an interplay between chromatin accessibility and meiotic recombination that we are just beginning to understand. To date, the majority of insights into the genomic distribution of meiotic CO events have come from the sequencing of large natural populations or from artificially created *F*_2_, backcross and/or recombinant inbred lines. Such approaches can require several generations of plant work, and always involve dealing with a large number of progeny plants. Therefore, the application of novel sequencing and bioinformatic approaches for the direct detection of meiotic recombination in any given hybrid plant is of major utility as it will facilitate rapid recombination profiling in any given environmental context.

**Fig. 1:**
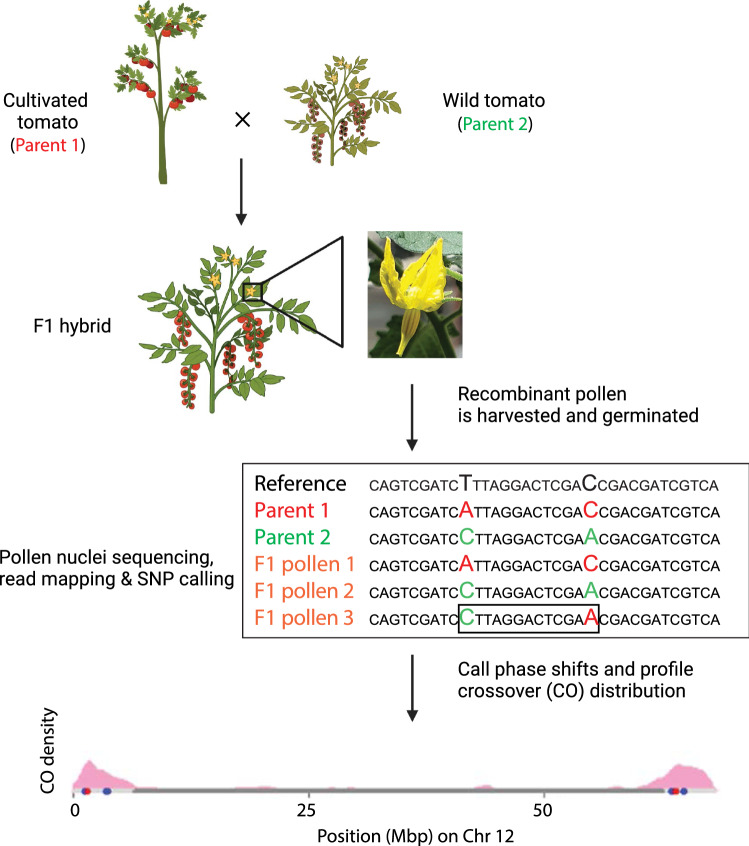
Pollen nuclei sequencing to profile meiotic recombination in tomato hybrids. From top to bottom, a cultivated tomato (Parent 1) is pollinated by a wild tomato (Parent 2). The hybrid progeny is grown and pollen harvested from open flowers. A photo of a *S. lycopersicum* x *S. cheesmaniae* flower is shown as an example. Germination of pollen grains is carried out in vitro to allow for the extraction of pollen nuclei. High molecular weight DNA can be extracted from pollen nuclei and used for the generation of DNA sequencing libraries. DNA sequence reads, from both parents and recombinant pollen, are mapped against a reference genome sequence and used for SNP calling. The mapped reads can be used to call phase shifts, estimate crossover (CO) positions at high precision, and subsequently to generate chromosomal CO distributions. This figure was created with BioRender.com

Taking advantage of developments in linked read sequencing and genome bioinformatics, cost-effective high-throughput recombination profiling has successfully been applied on pollen nuclei in Arabidopsis, tomato and apricot (Sun et al. 2019; Rommel Fuentes et al. 2020; Campoy et al. 2020) (Fig. 1). The ability to detect phase block shifts signifying CO recombination is limited by the availability and density of segregating markers (including SNPs and SVs), the length of DNA fragments that can be obtained from recombined chromosomes, and the intrinsic error rate of the sequencing technology used. Illumina sequencing based profiling of pollen nuclei is a feasible approach that satisfies these requirements (Sun et al. 2019; Rommel Fuentes et al. 2020). Besides the low amount of large sized input DNA required and high base call quality output, a substantial number of individual pollen nuclei can be typed, each with a unique recombination profile. The unambiguous read mapping and accurate identification of haplotype phases by this approach has thus led to the identification of phase block shifts in recombinant tomato pollen marking COs and GCs (Fig. 1) (Rommel Fuentes et al. 2020). Using pollen profiling in tomato, CO recombination has been assessed rapidly and cheaply with precision down to a resolution of 2 bp (Rommel Fuentes et al. 2020). Such a high resolution enables the detection of several types of recombination events such as illegitimate recombination and gene conversions both in euchromatin and heterochromatin domains. An additional advantage is that hundreds of recombination events from a large number of recombinant genome copies can be obtained from a single bulked pollen sample. COs and GCs pinpointed at the SNP resolution level have provided further insight into gross similarities of recombination. Moreover, pollen profiling may be applied to reliably profile meiotic recombination in a wide variety of crop species, including in crops with relatively long generation periods, without the laborious and time-consuming production and screening of offspring populations, greatly benefitting introgression hybridization and precision breeding. A complementary approach to sequencing of pollen DNA is the targeted measurement of meiotic recombination rates in pollen nuclei using Crystal Digital PCR, as recently established in barley (Ahn et al. 2021). Ultimately, genome-wide and targeted high throughput recombination profiling in pollen will help discover features that determine recombination rates.

Although meiotic recombination profiles from bulked pollen can be used to assess recombination frequency, it however does not permit genetic map construction, as an absolute number and position of COs per gamete cannot be retrieved from a bulked sample. Recently, single cell sequencing of haploid gamete genomes has been used to detect linked COs per gamete, facilitating genetic map construction and subsequent chromosome-level, phased, assemblies for a 242 Mbp diploid apricot genome (Campoy et al. 2020). The method involves phasing of gamete genomes in a hybrid genotype without having any information of the parents of the hybrid. Essentially, short reads from pollen nuclei are aligned to PacBio-based genome assemblies of the hybrid genotype to detect haplotype specific SNP markers, phase shifts and, thereby, meiotic CO. The recombination frequency (cM/Mb) for each recombinant pollen nucleus is subsequently used for genetic map construction (Campoy et al. 2020) It will be interesting to see whether this approach can be effectively applied for CO profiling and subsequent genetic map construction of hybrid tomatoes.

The pollen nuclei recombination profiling results described to date were obtained with 10× genomics based linked-read DNA sequencing technology (Sun et al. 2019; Rommel Fuentes et al. 2020; Campoy et al. 2020). Notably 10 × genomics have recently phased out approaches relating to genomic DNA sequencing including the “Chromium Genome Reagent” and “Chromium Single Cell CNV” kits due to patent dispute issues. In future it will be important to explore alternative linked-read (e.g., TELL-seq and stLFR) and single cell sequencing approaches (e.g,. scRNA-seq) to ensure sequencing based recombination profiling of pollen nuclei will be possible in the future (Chen et al. 2019, 2020; Wang et al. 2019; Chiu et al. 2022).

## Exploiting modern genome editing and tomato cultivation approaches to expedite meiosis research in tomato

Genome editing approaches have democratized reverse genetic studies in genetically transformable plant species, and this has facilitated an increase in the availability of meiotic mutants in a wide array of plant species (Wang et al. 2021). Tomato is no exception—a number of studies have employed genome editing to generate DNA repair and meiotic mutants in tomato (Mieulet et al. 2018; de Maagd et al. 2020; Whitbread et al. 2021). Through the detailed characterization of mutants in meiotic gene homologues in divergent plant species, and therefore different epistatic contexts, it will be possible to develop a more rounded view of meiotic gene function.

The comparison of RTR (RECQ/TOP3α/RMI) DNA repair complex mutants in Arabidopsis and tomato has revealed different phenotypes in the two species and represents an important example of the power of comparative genetic studies. The RTR complex, conserved in eukaryotes, is involved in the dissolution of Holliday junction-like recombination intermediates and favors NCO DNA repair pathways in both somatic and meiotic contexts (Hatkevich and Sekelsky 2017). The three complex members can work together (RECQ as helicase, TOP3α as topoisomerase and RMI as a structural protein) and independently (Emmenecker et al. 2022). Arabidopsis mutants that are null for RECQ4 helicase activity increase meiotic CO through the class II CO pathway (Séguéla-Arnaud et al. 2015). Likewise the mutation of the tomato *RECQ4* gene in an intraspecific (*S. lycopersicum* x *S. lycopersicum*) mixed background and an interspecific (*S. lycopersicum* x *S. pimpinellifolium*) hybrid leads to increased meiotic CO (Mieulet et al. 2018; de Maagd et al. 2020). In contrast to *recq4* mutants, Arabidopsis null *top3α* (Hartung et al. 2008; Dorn et al. 2018) and *rmi1* (Chelysheva et al. 2008; Hartung et al. 2008) mutants exhibit meiotic catastrophe as they are crucial for promoting recombination intermediate resolution. Specific Arabidopsis *top3α* (Séguéla-Arnaud et al. 2015) and *rmi1* (Séguéla-Arnaud et al. 2017) mutants that lack C-terminal OB2 and Zinc Finger domains also exhibit increased meiotic CO, and are therefore separation-of-function mutants. In tomato null *top3α* mutants are embryo lethal, indicating a crucial role in early development, whereas predicted null *rmi* mutants do not have a highly disturbed meiosis and can produce pollen, fruits and seeds (Whitbread et al. 2021). The different phenotypes of RTR complex mutants between Arabidopsis and tomato remain to be fully explained and highlight that further meiotic mutant generation in tomato will likely provide insights into the evolution of meiotic gene function.

**Fig. 2:**
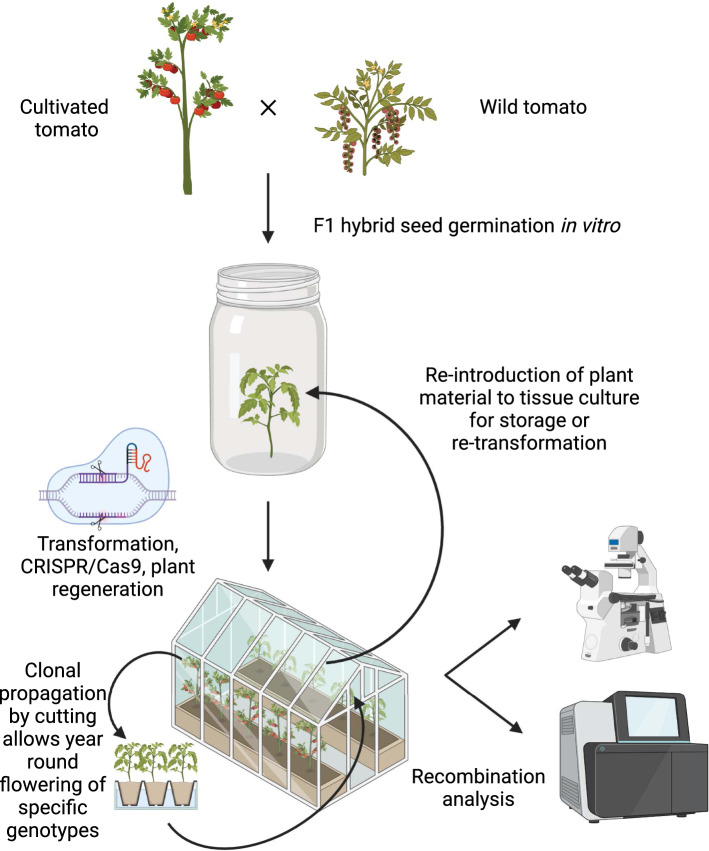
Genome editing and cultivation methods that make tomato an ideal meiotic model. From top to bottom, a cultivated tomato is pollinated by a wild tomato, and hybrid seeds can be germinated in vitro. Non-expanded, cut, leaves from 4–5-week-old plants are used as explants for infection with *Agrobacterium* strains harboring CRISPR/Cas9 constructs targeting meiotic genes of interest. Selection by antibiotic resistance allows for isolation of putative transgenic shoots which are used to generate rooted plantlets. Rooted, PCR-validated, transgenic plants can be transferred to soil. Flowering plants can be checked for fertility traits (including pollen viability, fruit setting and seed production) and meiotic recombination can be analyzed through cytological analysis of meiotic chromosome spreads by microscopy or through sequencing of recombinant gametes/offspring (see also Fig. 1). Clonal propagation through cuttings allows specific tomato genotypes to be maintained indefinitely and to be continuously flowering. Tomato genotypes can also be re-introduced into tissue culture by sterilizing early lateral shoots and propagation on rooting media. This figure was created with BioRender.com

The availability of meiotic stage buds and flowers is important in meiosis research as these are the main tissues of interest for practical experimentation. Numerous dwarf, rapid-flowering tomato varieties are available, including Micro-Tom (Meissner et al. 1997), and are suitable for studying tomato meiosis and reproduction. In ideal growth conditions Micro-Tom flowers 5 weeks after sowing, which is similar to Arabidopsis, and Micro-Tom can be easily genetically transformed via the agrobacterium method (Meissner et al. 1997; Sun et al. 2006). Further to this, through the propagation of tomato plants by cutting lateral shoots and rooting in soil, it is possible to maintain specific tomato genotypes indefinitely (Fig. 2). Clonal propagation by cutting facilitates continuous availability of tomato buds, flowers and pollen, of any genotype, for performing cytology, sequencing or crossing experiments. The continuous availability of tomato flowers is advantageous compared to other crop species used as meiotic models. For examples most maize and rice genotypes take at least 2 months to reach maturity and meiotic buds are available for a short window of time, typically one to two weeks. Constant flowering of tomato genotypes is possible in both determinate (e.g., Micro-Tom) or indeterminate (e.g., Moneymaker or Moneyberg-TMV) tomato cultivars but requires slightly different cultivation practices. Almost all natural, wild and greenhouse tomato lines are indeterminate. Indeterminate tomatoes are essentially perennial plants that develop into vines that continue to grow almost indefinitely when sufficient nutrients and appropriate environment is provided. Therefore, propagation of indeterminate tomatoes by cuttings does not need to be carried out very frequently to ensure flower availability, whereas determinate tomato genotypes that carry the *self-pruning* (*sp*) mutation (Pnueli et al. 1998) senesce much quicker and therefore cuttings must be carried out on a regular basis to ensure flowers are available. Appropriate phytosanitary practice is important when working with tomato plants due to the susceptibility of many cultivated and wild tomatoes to various pests and pathogens. Notably almost all tomato accessions are fully susceptible to the Tomato brown rugose fruit virus (ToBRFV) which was first discovered in 2015 in Jordan and has since caused major losses for tomato producers around the world (Salem et al. 2016; Zinger et al. 2021). As such the exchange of tomato plant materials is completely prohibited between some countries, and even when permitted seed exchange requires phytosanitary documentation, which currently represents a major challenge for the tomato research community.

Another possibility in the tomato system is that lateral shoots taken from plants grown in soil can be sterilized by mild bleach treatment and then re-introduced to in vitro culture (Fig. 2). This allows specific tomato genotypes (including fully sterile meiotic mutants) to be expanded by further cutting in vitro where they can be stored for a longer period of time at lower temperature (e.g., 14 degrees centigrade) or for genetic transformation experiments. We propose that the afore-mentioned tomato cultivation methods have great potential to expedite meiosis research in tomato and wild relatives.

## Emerging themes and future directions

Several outstanding questions on meiotic recombination related to (peri)centromeres, genetic polymorphism and temperature are now ripe to be addressed in tomato and wild relatives.

The identification of *cis* and *trans* factors that contribute to low recombination in plant (peri)centromeres could be readily carried out in tomato. Roughly 65% of the physical length of tomato chromosomes do not recombine during meiosis (Demirci et al. 2017; De Haas et al. 2017; Rommel Fuentes et al. 2020). As such tomato is a suitable model to understand how the recombination machinery is skewed towards the two distal ends of the chromosomes, while the (peri)centromeric regions are cold spots. It will be important to establish in future if low-recombination in (peri)centromeric regions is due to modifications to chromatin, due to yet-to-be-identified genetic pathways, or a combination of both.

Another relevant topic to explore in tomato is the role of genetic polymorphism in determining chromosomal landscapes of meiotic recombination. A recent study indicates that genetic polymorphism may not be the key driver of broad recombination patterns in intraspecific Arabidopsis hybrids (Lian et al. 2022). The cultivated tomato can be crossed with twelve related species, representing more than 2 million years of genetic divergence (Bedinger et al. 2011; Pease et al. 2016). The generation of high-resolution CO maps in this set of hybrids will help unravel how a range of genetic polymorphism frequencies affect meiotic recombination number and distribution in interspecific hybrids. Building on previous machine learning approaches that have identified AT-rich sequence, DNA shape and chromatin accessibility as major contributors to recombination profiles (Demirci et al. 2018; Lian et al. 2022), we propose that recombination data from such a series of tomato hybrids could be further explored with machine learning. Together with the analysis of many reference-grade tomato genomes, CO profiles from recombinant inbred lines, historical recombination rate, and epigenetic modifications deeper insights into recombination in tomato could be made (Aflitos et al. 2014; Demirci et al. 2017; Alonge et al. 2020; Wang and Baulcombe 2020; Rengs et al. 2022; Zhou et al. 2022; Fuentes et al. 2022). In addition to this analysis, genome editing directly in hybrid tomato genotypes will likely prove to be informative. Tomato hybrids tend to respond well to tissue culture and genetic transformation (de Maagd et al. 2020; van Rengs, Wang and Underwood, unpublished observation), opening up avenues for direct modulation of meiotic factors in hybrid plants.

Meiosis and meiotic CO rate are sensitive to temperature in a series of plant species, including Arabidopsis and barley (Higgins et al. 2012; Phillips et al. 2015; Lloyd et al. 2018; Modliszewski et al. 2018). The further study of this topic in a wider array of plants is gathering interest due to climate change and the more frequent occurrence of extreme temperatures (Arnell et al. 2019). In most crops thermosensitivity is especially prominent during the reproductive phase, with high temperature negatively influencing pollen viability and fertilization leading to decreased crop production (Hatfield et al. 2011). This is also evident in tomato, where lines with pollen thermo tolerance and sensitivity have been identified (Paupière et al. 2017). The response of meiotic CO rate in cultivated tomato, or hybrids with wild relatives, has yet to be studied. Modern tomato breeding has mainly focused on a limited set of traits including higher productivity, increased sensory and nutritional value, adaptation to different cultivation systems, and resistance traits (Bai and Lindhout 2007; Schouten et al. 2019). As a result of this focus, the genetic basis of tomato cultivars was severely narrowed, known as the ‘domestication syndrome’ (Hammer 1984; Doebley et al. 2006; Bai and Lindhout 2007; Bauchet and Causse 2012), although in the last two decades breeders have begun to exploit wild germplasm (Schouten et al. 2019). Nonetheless, the relatively small genetic variation in tomato has become apparent in the face of rapidly changing environmental conditions. While these challenges push breeding efforts towards better biotic and abiotic stress tolerance, the reduced genetic variation that resulted from extensive inbreeding has decelerated crop improvement (Sourdille and Devaux 2021). To widen the genetic basis, the further exploration and exploitation of wild germplasm may be able to counteract the sensitivity of tomato meiosis and reproduction to temperature. Specifically, wild species closely related to tomato display great diversity in terms of growth habit, habitat and morphology, ranging from perennial species that grow in wet rainforests to annual herbs in deserts (Knapp 2002). Therefore, cultivated tomato and wild relatives are suitable species for exploring the influence of temperature on meiosis because pollen production is possible over a very wide range of temperatures (from 20 to 36 °C, with an optimum of about 26 °C). We propose that the application of pollen profiling at different temperatures could unravel the sensitivity of tomato meiosis to temperature.

Here, we outlined several resources and modern approaches that can be used in the future study of meiosis in tomato and wild relatives. More than being an important fruit crop, the wide diversity of natural and artificial tomato genetic resources together with the burgeoning possibilities brought about by genome editing and third generation sequencing suggest that the tomato model can play an important role in the fertile field of plant meiosis research. We conclude that tomato represents a suitable model system to address outstanding problems on recombination suppression, the impact of genetic heterozygosity on CO landscapes and the sensitivity of meiosis to abiotic stress.
